# Mechanics of the cellular microenvironment as probed by cells in vivo during zebrafish presomitic mesoderm differentiation

**DOI:** 10.1038/s41563-022-01433-9

**Published:** 2022-12-28

**Authors:** Alessandro Mongera, Marie Pochitaloff, Hannah J. Gustafson, Georgina A. Stooke-Vaughan, Payam Rowghanian, Sangwoo Kim, Otger Campàs

**Affiliations:** 1 Department of Mechanical Engineering, University of California, Santa Barbara, CA USA; 2 Biomolecular Science and Engineering Program, University of California, Santa Barbara, CA USA; 3 Center for Systems Biology Dresden, Dresden, Germany; 4 Cluster of Excellence Physics of Life, TU Dresden, Dresden, Germany; 5 Present Address: Department of Pathology, Brigham and Women’s Hospital and Department of Genetics, Harvard Medical School, Boston, MA USA

**Keywords:** Biophysics, Developmental biology, Mechanotransduction

## Abstract

Tissue morphogenesis, homoeostasis and repair require cells to constantly monitor their three-dimensional microenvironment and adapt their behaviours in response to local biochemical and mechanical cues. Yet the mechanical parameters of the cellular microenvironment probed by cells in vivo remain unclear. Here, we report the mechanics of the cellular microenvironment that cells probe in vivo and in situ during zebrafish presomitic mesoderm differentiation. By quantifying both endogenous cell-generated strains and tissue mechanics, we show that individual cells probe the stiffness associated with deformations of the supracellular, foam-like tissue architecture. Stress relaxation leads to a perceived microenvironment stiffness that decreases over time, with cells probing the softest regime. We find that most mechanical parameters, including those probed by cells, vary along the anteroposterior axis as mesodermal progenitors differentiate. These findings expand our understanding of in vivo mechanosensation and might aid the design of advanced scaffolds for tissue engineering applications.

## Abstract (web summary)

During mesodermal differentiation of living zebrafish embryos, individual cells probe the stiffness associated with the foam-like architecture of the tissue as a part of their mechanosensing responses.

## Main

Cells in tissues constantly make decisions based on the biochemical and mechanical cues they perceive in the local microenvironment^1–4^. Using well-controlled mechanical microenvironments in two-dimensional (2D) and three-dimensional (3D) cell culture systems (for example, hydrogel scaffolds, purified matrices or substrates with adjustable stiffness), it has been shown that different mechanical inputs affect cell behaviours in the absence of instructive biochemical cues, including cell proliferation, cell differentiation, stem cell maintenance and cell migration^1,5–10^, as well as tumour progression and metastasis^11^. Notably, stem cell differentiation can be guided by tuning the substrate stiffness^10^. Similarly, different threshold values in scaffold elasticity also regulate germ layer specification^12^, suggesting that in addition to adult tissue homoeostasis and regeneration, mechanical cues may be important to control cell fate during embryogenesis^1,2,13^. Like morphogens, spatial gradients of mechanical parameters in embryonic tissues could provide positional information to cells. Moreover, stage-specific changes in tissue mechanics could serve as trigger signals to drive specific cell behaviours, as recently shown in vivo for neural crest migration^14–16^.

The mechanics of the cellular microenvironment have also been shown to be key in bioengineering applications related to stem-cell-based tissue regeneration^1,17,18^. In this case, it is important to design synthetic scaffolds with controlled mechanical parameters that mimic the microenvironment that cells perceive in vivo during the regenerative response. Most studies have focused on the control of scaffold stiffness, as it has been shown to affect cell differentiation in vitro. However, living tissues are often more complex than linear elastic materials, with multiple structures (cytoplasm, cell cortex, adhesion at cell–cell contacts, extracellular matrix and so on) contributing to their mechanical response at different length scales and timescales^19^. Recent studies using synthetic hydrogel matrices with controlled viscoelastic properties have shown that the timescale of stress relaxation affects cell differentiation, among several other cell behaviours^8,20^. While it is now clear that multiple mechanical parameters influence cell behaviour in vitro, very little is known about the mechanical parameters of the microenvironment that cells perceive in vivo, the structures that cells mechanically probe and how these mechanical cues affect cell behaviour within developing 3D tissues. In particular, the mechanical cues that cells experience during embryogenesis, as differentiation of specialized structures takes place, are largely unknown.

**Fig. 1:**
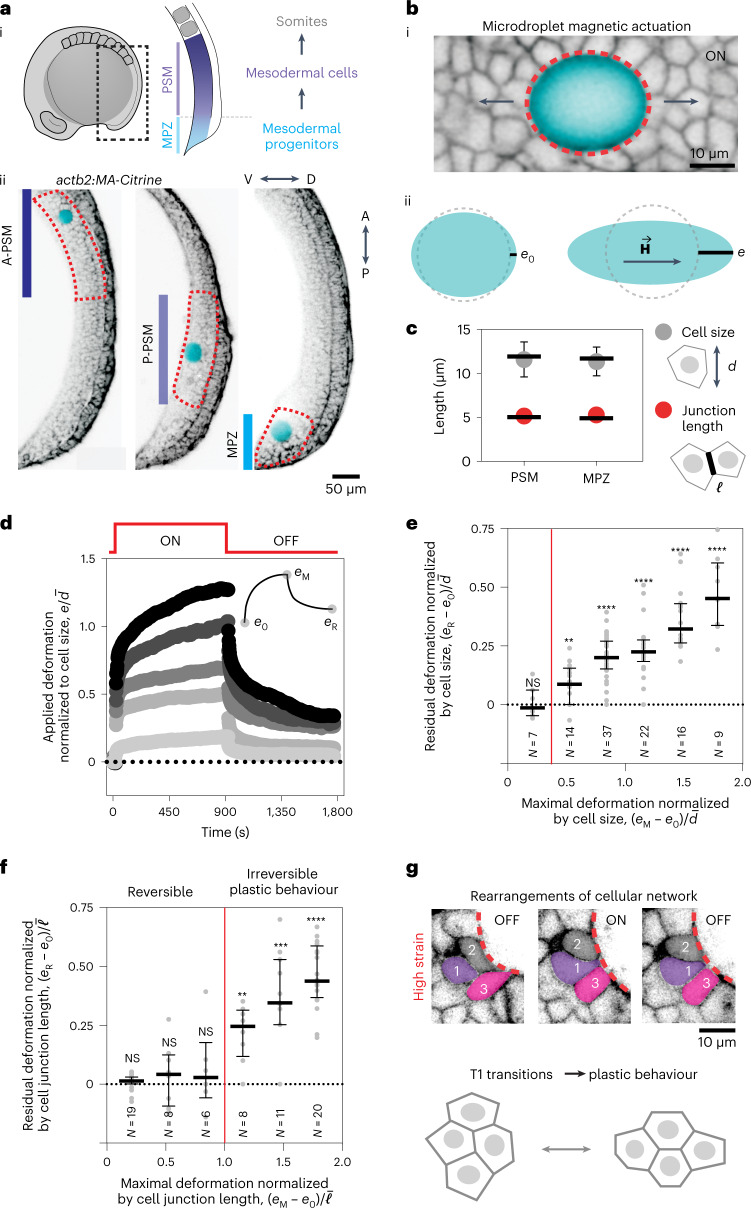
Junctional length establishes the onset of tissue plasticity. **a**, Sketch showing a lateral view of a ten-somite stage embryo highlighting the posterior region of the body (dotted black rectangle) where mesodermal progenitors progressively differentiate as they transit from the MPZ to the PSM (i). Confocal sections along the sagittal plane of posterior extending tissues in membrane-labelled *Tg(actb2:MA-Citrine)* embryos (inverted) containing ferrofluid droplets (cyan) in different regions along the AP axis, namely the A-PSM, the posterior PSM (P-PSM) and the MPZ (ii). V, ventral; D, dorsal; A, anterior; P, posterior. The red dashed contours highlight each region (A-PSM, P-PSM and MPZ). **b**, Confocal section of droplet (cyan) during actuation (magnetic field ON; membrane label, inverted; i). Red dashed line indicates droplet contour and arrows indicate the direction of forces applied by the droplet. Sketches defining the induced droplet elongation *e* along the direction of the applied magnetic field $\textbf{H}$ and the droplet pre-elongation before actuation, *e*_0_ (ii). Dashed lines indicate the unelongated droplet. **c**, Mean (dots) and median (lines) values of cell size (diameter, *d*; grey) and junctional length ($\ell$; red) in the MPZ and PSM, reanalysed from the literature^27^. The inset at the right shows illustrates the parameters. Error bars, s.e.m. **d**, Examples of the time evolution of droplet deformation (normalized droplet extension, $e/\bar d$, where $\bar d$ is the average cell size (diameter); induced strain) during actuation cycles (OFF–ON–OFF) for different values of the applied magnetic field, leading to varying *e*_M_ values. Both *e*_0_ and *e*_R_ are defined in the inset. **e**,**f**, Residual droplet elongation normalized by average cell size, $\left( {e_{\mathrm{R}} - e_0} \right)/\bar d$ (**e**) or by average junctional length, $\left( {e_{\mathrm{R}} - e_0} \right)/\bar \ell$ (**f**), for varying values of the applied maximal droplet elongation (*e*_M_ − *e*_0_) normalized by average cell size (**e**) or junctional length (**f**) in the posterior paraxial mesoderm. Vertical red lines in **e** and **f** indicate the onset of plastic, irreversible deformation. Median and interquartile range are shown. *P* values were obtained from one-sample two-tailed *t*-tests. NS, not significant; **P = 0.0025, ****P < 0.0001. **g**, Snapshots showing confocal sections of tissue next to a droplet (dotted red line) during an actuation cycle (OFF–ON–OFF) causing T1 transitions that lead to plastic changes in the local tissue architecture (bottom). Source data

During the formation of the vertebrate body axis, mesodermal progenitors located at the posterior end of the body (the mesodermal progenitor zone, or MPZ) progressively differentiate into mesodermal cells, establishing the presomitic mesoderm (PSM) and eventually organizing into somites^21^ (Fig. 1a(i)). In this process, cells modify their transcriptional profile^22^ and progressively acquire an epithelial-like phenotype through a process of mesenchymal-to-epithelial transition^23^. In contrast to amniotes, which feature a considerable amount of extracellular matrix (ECM) in the paraxial mesoderm^21,24,25^, zebrafish have little or no ECM between cells in such tissues^26^. The mechanical parameters perceived by cells in these zebrafish tissues are thus unlikely to be related to ECM stiffness. Recent in vivo mechanical measurements of the (nonlinear) tissue mechanical response associated with large tissue deformations (large strains) showed that zebrafish posterior tissues undergo a jamming transition from a fluid-like state in the MPZ to a solid-like state in the PSM^27^, consistent with their foam-like tissue architecture. However, the (linear) mechanical response of the same tissues to small deformations (small strains) has been shown to be viscoelastic and display spatial variations in the tissue^28^. These experiments revealed a complex mechanical landscape, with different mechanical response at small versus large applied strains (plasticity) and a time-dependent response for small tissue deformations (viscoelasticity). In complex materials with strain-dependent and time-dependent material properties, the perceived mechanical parameters depend both on the strain and timescales at which the material is mechanically probed^19,29^. The observed foam-like architecture of posterior tissues during body axis elongation is very different from the polymeric gel-like microenvironments (ECM or synthetic gels) used in vitro to study how cells sense the microenvironment mechanics at molecular scales^30–35^. It is unclear how cells probe such a different microenvironment in vivo and what the mechanical parameters of the tissue structure are that they perceive.

## Cell rearrangements control the onset of plasticity

In polymeric materials (ECM or cytoskeletal structures), plastic behaviour is controlled by irreversible events at molecular scales. By contrast, plasticity in foam-like materials is associated with rearrangements of the cellular structure. To understand what structures control plastic deformations in posterior zebrafish tissues during axis elongation, we characterized the magnitude of the deformations (or strains) that mark the transition from a linear viscoelastic response to a plastic regime. To do so, we employed magnetically responsive microdroplets embedded in the paraxial mesoderm at different locations along the anteroposterior axis^27,28^. By applying a constant, uniform magnetic field to droplets previously inserted between the cells in the tissue (Fig. 1a(ii) and Methods), we induced ellipsoidal droplet deformations characterized by an elongation *e* = *b* − *R* in the direction of the applied magnetic field $\textbf{H}$ (Fig. 1b(i),(ii))^28,36^, with *b* and *R* being the droplet semi-axis along the direction of the magnetic field and the radius of the undeformed droplet, respectively. To contextualize droplet deformations in a cellular, foam-like tissue architecture, we normalized the induced droplet elongation, *e*, with the average cell diameter *d* (Fig. 1c and Methods), namely *e*/*d*, which characterizes the applied strain in the material. Increasing magnetic field intensities led to larger droplet deformations (and strains), up to approximately two cell diameters (or 200% strain; Fig. 1d,e). Upon actuation (magnetic field ON; Fig. 1d), droplets progressively elongated from their initial predeformed state, characterized by *e*_0_, and reached their maximal elongation *e*_M_ just before turning off the magnetic field at time *t* = 15 min (Fig. 1b(ii),d). After removing the magnetic field (magnetic field OFF; Fig. 1d), capillary stresses pulled the droplet back towards its undeformed, spherical state, progressively reducing the droplet elongation. For values of the applied deformation (*e*_M_ − *e*_0_) below a threshold value of approximately half the cell size ((*e*_M_ − *e*_0_)/*d* ≈ 0.45; Fig. 1e), the process was reversible and the droplet relaxed back to the initial state. By contrast, when the maximal applied deformation (*e*_M_ − *e*_0_) was above this threshold, the droplet did not fully relax, displaying instead a residual elongation *e*_R_ that quantifies the degree of irreversible (plastic) deformation (Fig. 1e). These results show the existence of a threshold length scale to induce irreversible changes. Normalizing applied deformations with the average cell–cell junction length $\bar \ell$ shows that the onset of plastic behaviour occurs when applied deformations exceed the cell–cell junction length (Fig. 1c,f), indicating that the characteristic length scale controlling irreversible (plastic) tissue rearrangements is the cell–cell junction length and suggesting that tissue plasticity is associated with cell rearrangements. Indeed, large enough applied deformations induced permanent cell rearrangements in the neighbourhood of the droplet (Fig. 1g). This result can be interpreted as the existence of a yield strain ($\left( {e_{\mathrm{M}} - e_0} \right)/\bar \ell \approx 1$; Fig. 1f) in the tissue associated with its foam-like architecture, consistent with previously reported nonlinear tissue mechanics^27^.

## Cells probe the linear mechanical response of the tissue

**Fig. 2:**
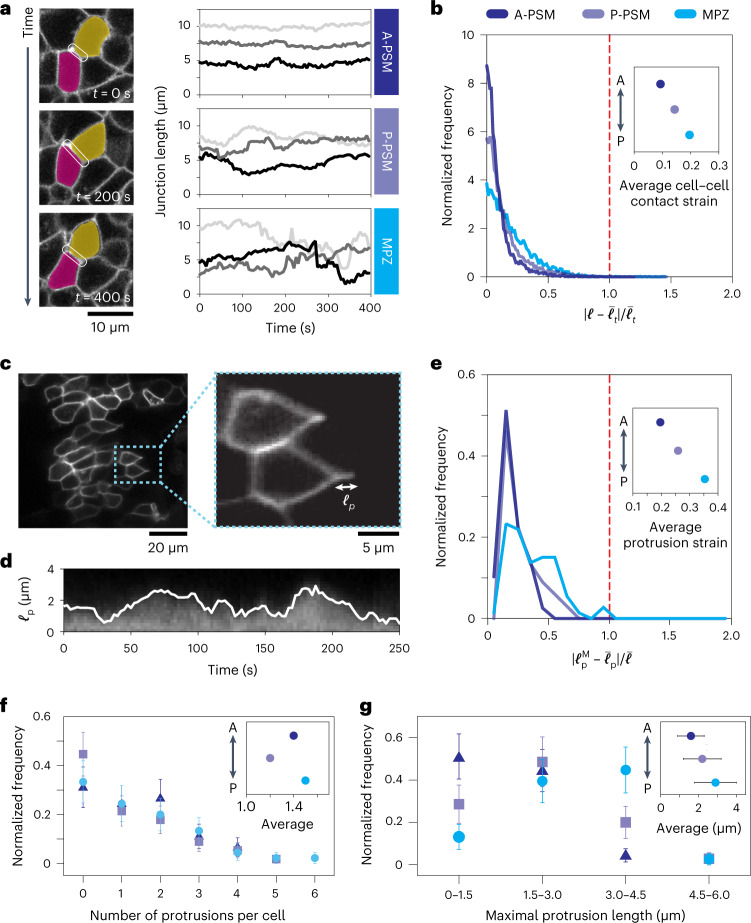
Cells endogenously probe the linear mechanics of the tissue. **a**, Confocal sections showing the temporal changes in cell junction length (white outline) over 400 seconds, and time traces of junction length for cells in different regions of the tissue, showing that junction length is less variable in the A-PSM than in the MPZ. **b**, Normalized frequency (distribution) of the magnitude of relative variations in junction lengths $\left| {\ell - \bar \ell _t} \right|/\bar \ell _t$ (endogenous applied strain at cell junctions) in the A-PSM and P-PSM (*N* of approximately 3,500 junctions in each region; four embryos), as well as the MPZ (*N* = 7,896; three embryos). The average endogenous applied strains at cell junctions (inset) are much smaller than the yield strain in the tissue (red dotted line). **c**, Confocal sections of PSM tissue in mosaic membrane-labelled embryos showing cell protrusions between cells. The length of each protrusion $\ell _{\mathrm{p}}$ (inset) can be measured at each time point. **d**, Kymograph showing the fluorescence intensity along a protrusion path, enabling the determination of the time evolution of the protrusion length (white line), $\ell _{\mathrm{p}}(t)$. **e**, Normalized frequency of maximal protrusion strains $\left| {\ell _{\mathrm{p}}^{\mathrm{M}} - \bar \ell _{\mathrm{p}}} \right|/\bar \ell$ in the different regions. Average protrusion strains (inset) are largest in the MPZ but much smaller than the yield strain in the tissue (red dotted line). Number of protrusions, *N* = 78 (A-PSM), 67 (P-PSM) or 73 (MPZ). **f**,**g**, Normalized frequency of the number of protrusions per cell (**f**) and their maximal lengths (**g**), with the average protrusion number per cell and average protrusion length shown in the insets of **f** and **g**, respectively. Mean ± s.d.; in **f**, number of cells *N* = 45 (A-PSM), 56 (P-PSM) or 45 (MPZ); number of protrusions *N* = 62 (A-PSM), 67 (P-PSM) or 66 (MPZ); in **g**, number of protrusions *N* = 45 (A-PSM), 35 (P-PSM) or 38 (MPZ). Source data

The mechanical response of the tissue sharply changes when induced deformations are larger than cell–cell contact lengths, indicating that cells will perceive a different mechanical landscape depending on the endogenous strains that they actively generate in the tissue. Since both cell–cell junctions and cellular protrusions can actively generate forces to probe the cellular microenvironment^37–39^ and are mechanosensitive^40,41^, we quantified the strain levels that each of these structures generates. To characterize the strain level generated at cell–cell junctions in the tissue, we monitored the time evolution of junctional lengths $\ell$ (Fig. 2a) and quantified the endogenous strains by measuring their relative changes over time, namely $\left( {\ell - \bar \ell _t} \right)/\bar \ell _t$, with $\bar \ell _t$ being the time average for individual junctions (Methods). The measured distributions of the magnitude of endogenous junctional strains $\left| {\ell - \bar \ell _t} \right|/\bar \ell _t$ showed that progenitor cells in the MPZ generate larger strains than the more anterior PSM cells (Fig. 2b). However, in all cases, the average values of the endogenous strains at cell junctions, which range from 8 to 20% (Fig. 2b), were below the yield strain (Fig. 1f). In order to quantify the strains generated by protrusions between cells, we performed mosaic labelling of cell membranes and monitored each protrusion length, $\ell _{\mathrm{p}}$, over time (Fig. 2c,d and Methods). Tracking protrusions between cells and calculating the maximal strain levels of protrusions, namely $\left| {\ell _{\mathrm{p}}^{\mathrm{M}} - \bar \ell _{\mathrm{p}}} \right|/\bar \ell$ (with $\ell _{\mathrm{p}}^{\mathrm{M}}$ and $\bar \ell _{\mathrm{p}}$ being the maximal and average protrusion lengths; Methods), showed that protrusion strains were considerably larger in the MPZ than in the PSM (Fig. 2e). While cells generated approximately the same number of protrusions everywhere along the anteroposterior (AP) axis (Fig. 2f), the protrusions in the MPZ were consistently longer (Fig. 2g). Yet, all protrusion strains were below the yield strain (Fig. 2e). Altogether, this analysis indicates that strains at cell–cell contacts and those generated by protrusions in between cells can probe only the linear mechanical response of the tissue.

## Spatiotemporal characteristics of linear tissue mechanics

**Fig. 3:**
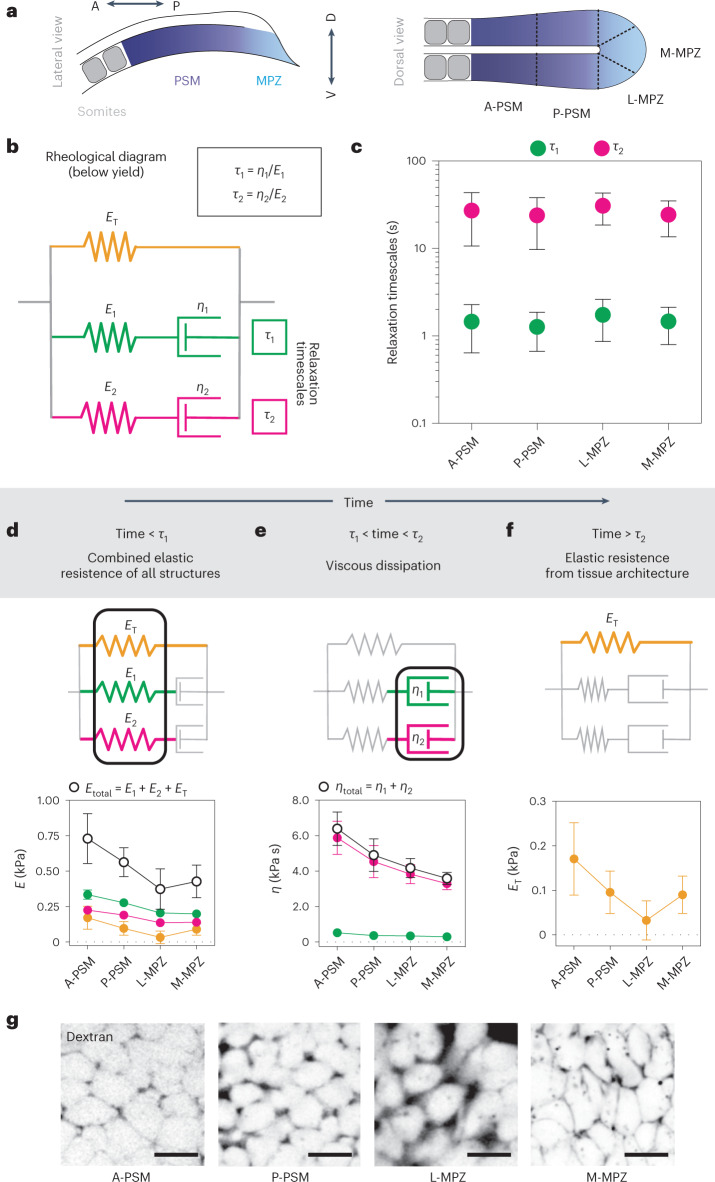
Stress relaxation timescales and time-dependent (linear) mechanical properties of the microenvironment along the AP axis. **a**, Sketch of lateral and dorsal views of the posterior body axis, showing the regions of the tissue in which measurements of linear material properties were performed. The PSM is divided into the anterior and posterior parts, A-PSM and P-PSM, respectively, and the MPZ is divided into the medial and lateral parts, M-MPZ and L-MPZ, respectively. **b**, Rheological diagram representing the tissue mechanical response below yield, with the stiffnesses (*E*_T_, *E*_1_, *E*_2_) and viscosities (*η*_1_, *η*_2_) for each branch of the rheological diagram shown. Two Maxwell branches, characterized by independent stress relaxation timescales *τ*_1_ and *τ*_2_, are in parallel with an elastic element accounting for the stiffness associated with the supracellular foam-like tissue architecture below yield. **c**, Measured values of the two relaxation timescales in the different tissue regions along the AP axis. Mean ± s.d. **d**–**f**, Mechanical properties of the microenvironment at different timescales, highlighting the relevant mechanical parameters of the rheological diagrams at each timescale regime (top). All components of the stiffness *E* and viscosity *η* are shown (colour coded) and the total added values are shown in black. All mechanical parameters display a posterior-to-anterior increasing gradient, with the exception of the stiffness of the supracellular tissue architecture, which displays a minimal value in the lateral MPZ. *N* = 15 for A-PSM, 11 for P-PSM, 17 for L-MPZ and 32 for M-MPZ. Plots show mean ± s.e.m. **g**, Confocal sections revealing the extracellular spaces between cells (fluorescent Dextran; inverted; Methods) in the different regions of the tissue along the AP axis. Scale bars, 10 µm. Source data

To understand the tissue mechanical parameters that cells probe, we quantified the tissue mechanical response at strain levels similar to those endogenously applied by cells (~5–30%). Using magnetically responsive oil droplets, we performed local creep experiments in the different regions of the paraxial mesoderm, as previously established^28^ (Figs. 1d and 3a and Methods). The observed droplet (strain) relaxation dynamics in the studied time period (0–15 min) could be properly accounted for by two distinct relaxation timescales associated with dissipation (friction) processes, as previously reported^28^. To describe the rheological response below yield (no plastic behaviour), we used a generalized Maxwell model (Fig. 3b) composed of two Maxwell (viscoelastic) branches characterized by independent relaxation timescales *τ*_1_ and *τ*_2_, in parallel with an elastic element that accounts for the elastic resistance of the supracellular tissue architecture below yield (before cell rearrangements—plastic events—occur). This is in contrast to previous work^28^, which was unaware of the existence of a yield strain in the tissue and did not account for the existence of such elastic behaviour of the tissue architecture below yield. Measurement of the two stress relaxation timescales showed that these were very different in magnitude, but both uniform along the AP axis (Fig. 3c). The shortest timescale, *τ*_1_, was approximately of 1.6 s, similar to the values previously obtained in vitro for the cytoplasm of cells in culture (0.1–1 s, depending on cell type and conditions^42^) and the stress relaxation timescale of the cytoplasm in the blastomeres of living zebrafish embryos (~2 s (ref. ^28^)). The other stress relaxation timescale, *τ*_2_, was approximately 25 s, over tenfold larger than the shortest (Fig. 3c) and similar to previously measured stress relaxation timescales of cellular junctions in epithelial monolayers in vivo (~20 s (ref. ^43^)). These results show that for deformations below the yield strain, the timescale over which stresses in the tissue relax (dissipation timescale) is approximately 25 s.

**Extended Data Fig. 1:**
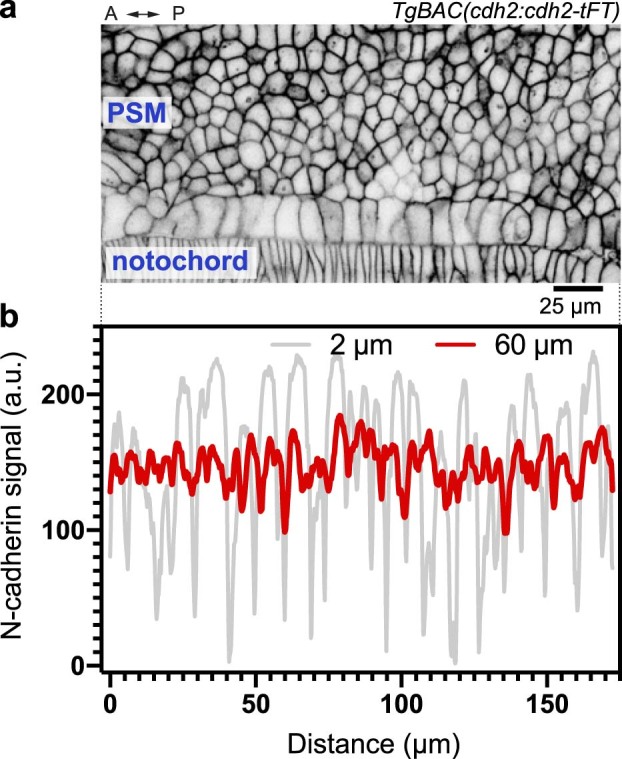
N-cadherin distribution in the paraxial mesoderm. **a**, Confocal section through the paraxial mesoderm (dorsal view) of *TgBAC(cdh2:cdh2-tFT)* embryos showing the N-cadherin (cdh2) distribution in the PSM tissue (notochord is also shown). **b**, Measured N-cadherin signal along the AP axis. Both the average signal obtained in a 2 *μ*m thick band (to visualize cell-to-cell variation along the AP axis; gray) and in a 60 *μ*m thick band (to average out cell-to-cell variations along the AP axis; red) along the paraxial mesoderm are shown.

To reveal the linear mechanical response of the tissue at different timescales, we measured the different elastic and viscous elements that define the rheological response of MPZ and PSM tissues (Fig. 3a). Below the smallest stress relaxation timescale (<1 s; Fig. 3d), the tissue behaved elastically, with its stiffness ranging between 400 Pa and 800 Pa, depending on the tissue region. At intermediate timescales (between ~1–30 s; Fig. 3e), viscous dissipation occurred with two very distinct viscosities. The smallest viscosity, of approximately 80–150 Pa s, was associated with stress relaxation at short timescales (*τ*_1_), whereas the largest one, approximately of 4,000 Pa s, was linked to the longer relaxation timescale (*τ*_2_). Finally, above the largest stress relaxation timescale (*t* > *τ*_2_; Fig. 3f), the tissue behaved elastically, with stiffness values ranging between 30 Pa and 180 Pa depending on the region of the tissue. While at very long timescales (~1 hour in the MPZ and longer timescales in the PSM) the tissue flows due to plastic cell rearrangements^44^, our results indicate that at timescales of a few minutes (>30 s and <10 min, approximately), the tissue responds to deformations as an elastic material, with its stiffness arising from the resistance to deformations of the foam-like cellular packings that define the tissue architecture. In addition to the time-dependent linear mechanical response, all measured mechanical parameters (stiffnesses and viscosities) monotonically increased away from the posterior end of the body (Fig. 3d–f), with the exception of the stiffness *E*_T_ associated with tissue architecture (Fig. 3f). Instead, *E*_T_ displayed a minimal value in the lateral MPZ (L-MPZ), the region where mesodermal progenitors commit to a mesodermal fate^22^. In foam-like structures, this supracellular stiffness *E*_T_ is strongly (nonlinearly) affected by the amount of extracellular spaces^45^, which are directly related to the level of physical confinement experienced by cells. Inspection of the extracellular spaces using fluorescent Dextran (Methods) showed that the amount of extracellular spaces was maximal in the L-MPZ (minimal cellular confinement), where the supracellular stiffness displayed its smallest value, and minimal in the anterior PSM (A-PSM; maximal cellular confinement), where the supracellular stiffness was the largest (Fig. 3f,g), as expected for foam-like architectures in which the volume fraction of extracellular spaces controls the tissue stiffness^45^. While AP gradients in cortical tension, cell adhesion and cell size could also potentially generate AP gradients in tissue stiffness^45^, no AP gradients of cell size or cell-generated stresses have been observed in these tissues^27^, and we did not observe any AP gradient in neural cadherin (N-cadherin) levels either (Extended Data Fig. 1). These results show that the tissue mechanical response at the strain levels applied endogenously by cells is time dependent and varies with the location in the tissue.

## Cells actively probe the supracellular tissue stiffness

**Fig. 4:**
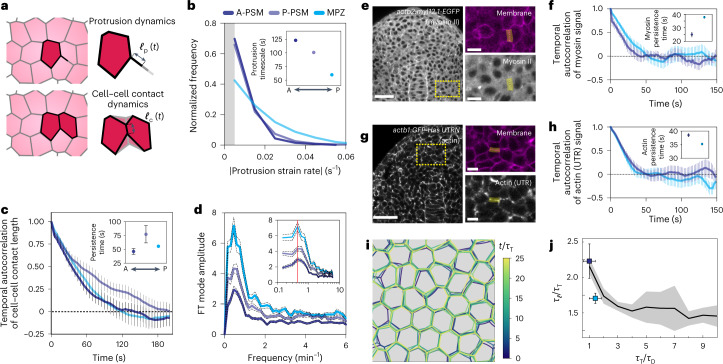
Characteristic timescales of endogenous mechanical probing of the cellular microenvironment. **a**, Sketches of cell protrusions and cell–cell junctions, indicating the measured time-dependent protrusion length and cell–cell junctional length, $\ell _{\mathrm{p}}(t)$ and $\ell _{\mathrm{c}}(t)$, respectively. **b**, Normalized frequency of the absolute value of the protrusion strain rates, showing minimal protrusion persistence timescales in the MPZ (inset). Limitations in protrusion tracking did not allow measurement of strain rates below approximately 0.005 s^−1^ (grey band). A-PSM (*N* = 1,490), P-PSM (N = 977) and MPZ (*N* = 391). **c**, Temporal autocorrelation of cell–cell junction length in different regions of the tissue along the AP axis, showing persistence (autocorrelation) timescales of approximately 1 minute (inset). Error bars, s.e.m. **d**, Fourier transform (FT) mode amplitudes of the time evolution of junction length in the tissue for different regions along the AP axis, showing a peak at frequencies (red line) of approximately 0.5 min^−1^. *N* of approximately 3,500 junctions in A-PSM and P-PSM (four embryos) and 7,896 junctions in MPZ (three embryos) in **c** and **d**. Error bars, s.e.m. **e**–**h**, Confocal sections of MPZ and PSM tissue regions in embryos with myosin II and membrane labels (**e**) and in embryos with actin (Utrophin, UTR) and membrane labels (**g**). Scale bars, 50 µm. Zoomed in yellow rectangular regions shown on right. Highlighted yellow region corresponds to analysis region around cell–cell contact. Scale bars, 10 µm. Measured myosin II (**f**) and actin (**h**) signal autocorrelations and the characteristic timescales for myosin II (**f**, inset) and actin (**h**, inset) dynamics. *N* = 12 and two embryos for each condition (MPZ, PSM, actin and myosin II). Error bars, s.e.m. (**e**,**f**). **i**, Simulated tissue dynamics arising from actomyosin-generated tension dynamics at cell–cell contacts (Methods). **j**, Predicted ratio of cell–cell contact length persistence timescale $\tau _\ell$ and tension persistence timescale *τ*_T_, in terms of the ratio between the tension persistence timescale *τ*_T_ and the dissipation timescale *τ*_D_. Measured values of these ratios in MPZ (light blue square) and PSM (violet square) are shown. Error band of simulation data (s.d.; *N* = 6 temporal correlations) and s.e.m. of experimental data are shown. Source data

Since the tissue material properties below yield are time dependent (viscoelastic), it is necessary to know the timescales at which cells mechanically probe their microenvironment to understand which mechanical parameters they perceive. Measuring the time evolution of protrusion strain rates, namely $\left( {1/\ell _{\mathrm{p}}} \right){\mathrm{d}}\ell _{\mathrm{p}}/{\mathrm{d}}t$ (Methods), we obtained their distribution in the different regions of the tissue (Fig. 4a,b). The characteristic timescale of protrusions, given by the inverse of the average strain rate, revealed that protrusions in the MPZ were the most dynamic, with characteristic timescales of approximately 1 min (Fig. 4b, inset). Protrusions in the PSM were slightly slower and displayed characteristic timescales of approximately 2 min (Fig. 4b, inset). We then characterized the characteristic timescale $\tau _\ell$ of variations in cell–cell junction length by measuring their persistence timescale, which we calculated from the autocorrelation function of the junctional length dynamics (Fig. 4a,c and Methods). The average persistence time was of approximately 1 min in all regions of the tissue (Fig. 4c, inset). Similarly, Fourier analysis of cell–cell contact length dynamics showed a characteristic timescale of 1–2 min, irrespective of the location of the cell in the tissue (Fig. 4d). To discern if the measured cell–cell contact length dynamics resulted from a passive response to stresses generated elsewhere or, by contrast, arose from actively generated forces at the cell–cell contact, we directly measured the dynamics of actin and non-muscle myosin II at cell–cell contacts (Fig. 4e–h and Methods). Both actin and myosin characteristic timescales (25–35 s; Fig. 4f,h) were consistently shorter than the measured cell–cell contact length dynamics (50–120 s; Fig. 4c,d). In order to relate the actomyosin-generated tension dynamics and the cell–cell contact dynamics, we performed simulations of tissue dynamics (Fig. 4i and Methods) and predicted the relations between the persistence timescale of cell–cell contact dynamics $\tau _\ell$, the characteristic timescale of actomyosin tension dynamics *τ*_T_ and the characteristic dissipation (or relaxation) timescale *τ*_D_ (Fig. 4j). While the dynamics of cell–cell contact lengths simply followed the tension dynamics for small dissipation values (*τ*_D_ ≪ *τ*_T_), when dissipation was considerable and *τ*_D_ ≈ *τ*_T_, the dynamics of cell–cell contact length became slower than the actomyosin tension dynamics, namely $\tau _\ell > \tau _{\mathrm{T}}$. This is because dissipation (friction) processes do not allow cell–cell contacts to change as fast as the tension does. Associating the tension dynamics timescale *τ*_T_ with the measured characteristic timescale of myosin dynamics at cell–cell contacts (Fig. 4f), and the dissipation timescale *τ*_D_ with the measured dissipation timescale below yield (*τ*_2_ in Fig. 3c), we found that the measured cell–cell contact persistence timescale followed the predicted behaviour in both the MPZ and the PSM (Fig. 4j). These results are consistent with cell–cell contact dynamics being actively driven by myosin-generated tension at cell–cell contacts, as previously suggested by the lack of correlation between the dynamics of different cell–cell contacts in these tissues^27^. While active processes drive mechanical probing, it is the resulting generated strains that deform the microenvironment and can probe its mechanics, as mechanical probing of any material requires deforming it. Our results reveal that cells actively probe their microenvironment, either via protrusions or contractions at cell–cell junctions, at timescales ranging between 1–2 min.

**Fig. 5:**
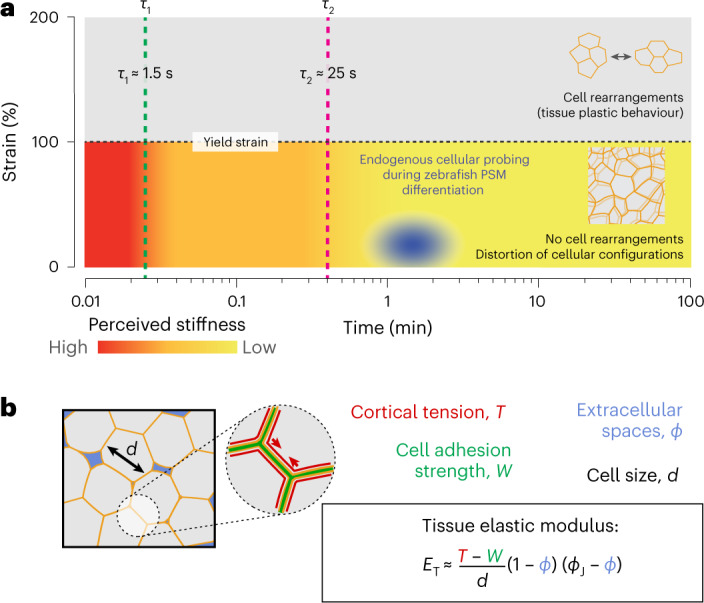
Cells probe the tissue stiffness associated with the local, foam-like architecture of the tissue. **a**, Schematic representation of the measured mechanics of the microenvironment at different strains and timescales during PSM differentiation. The blue ellipse represents the region mechanically probed by cells in these tissues, as indicated by our measurements (for length scales of >0.5 μm and timescales of >1 s). Above the yield strain, cell rearrangements (T1 transitions, shown in the inset) occur, leading to plastic (irreversible) remodelling of the tissue architecture. Below yield, the tissue maintains its local cellular configurations (no cell rearrangements; inset) and dissipates stresses at different timescales, *τ*_1_ and *τ*_2_. The perceived microenvironment stiffness below yield (colour coded) decreases over time as stresses are dissipated, eventually reaching a constant low value (yellow) associated with the elasticity of deforming cellular packing configurations. Active cellular probing of the microenvironment (blue ellipse) occurs at timescales (~1–2 min) longer than all microenvironment relaxation timescales and at small strains (~10–30%), indicating that during PSM differentiation, cells probe the stiffness associated with the local, foam-like tissue architecture. **b**, Cortical tension, *T*, cell adhesion, *W*, and the volume fraction of extracellular spaces, *ϕ*, affect the stiffness, *E*_T_, of the foam-like tissue at supracellular scales for timescales larger than the measured relaxation timescales (~30 s) and smaller than those leading to substantial T1 transitions (~30 min). The equation shows this relationship (*ϕ*_J_ being the jamming volume fraction for a disordered 3D foam), and the colours of the parameters in the equation correspond to the colours in the figure at the left.

Altogether, the results presented show that the mechanics of the microenvironment (below yield) display an effective stiffness that decreases over time, reaching a minimal value for timescales *t* > *τ*_2_ ≈ 25 s (Fig. 5a). Since cells actively probe their microenvironment at small strains (<35%; Fig. 1f; below yield strain) and timescales of approximately 1–2 min (Fig. 4b,c) in all regions of the tissue, our results are consistent with cells probing the tissue stiffness *E*_T_ associated with the local foam-like tissue architecture (Fig. 5a,b). Specifically, mesodermal progenitors probe the smallest stiffness (of ~30 Pa; Fig. 3f) in the L-MPZ (the region with least cellular confinement) as they differentiate into mesodermal cells, which in turn perceive increasing stiffness during their maturation in the PSM (up to 180 Pa; maximal cellular confinement; Fig. 3f). Since the stiffness *E*_T_ of a tissue with a foam-like architecture is directly related to the amount of extracellular space *ϕ* (Fig. 5b), these results suggest that cells may probe their degree of physical confinement, a parameter that has recently been shown to promote somatic-cell reprogramming in 3D environments through an accelerated mesenchymal-epithelial transition (MET)^46^.

## Outlook

Changes in both the stress relaxation timescales of the microenvironment or the timescale at which cells actively probe the tissue can make cells perceive different microenvironment mechanics, potentially causing changes in their behaviour, as reported in vitro^8^. While this study focuses on how cells probe the mechanical parameters associated with the tissue structure during zebrafish PSM differentiation, several in vitro studies have shown that cells can probe their microenvironment at molecular length scales and subsecond timescales^30,47,48^ beyond the spatial and temporal resolution of our experiments (approximately 0.5 μm and 1 s; Methods). Our results do not exclude the possibility that cells also probe their microenvironment at faster timescales and at molecular length scales. These small and fast active deformations could probe the mechanics of smaller subcellular structures. How much tissue-scale mechanics or the mechanics of subcellular structures contribute to regulating cell function remains to be determined in future work. Finally, in contrast to the case studied herein, tissues containing an ECM will be characterized by a different yield strain and additional stress relaxation timescales associated with ECM remodelling^21,25,49^. To understand what mechanical parameters cells might perceive in ECM-dominated microenvironments, it will be important to know the characteristics of active cellular probing in these contexts and how these compare to the strain-dependent and time-dependent mechanics of the microenvironment. In the case of amniote species^21,24,25^, the presence of an ECM between cells in the paraxial mesoderm, as well as the cell shapes in this region^50^, suggests that tissue mechanics may be dominated by the ECM and cells may therefore perceive the ECM stiffness. Future work will reveal if cells in different tissues, developmental stages or organisms probe similar tissue mechanical parameters in vivo as those reported here for zebrafish PSM differentiation.

Accurate knowledge of what mechanical parameters of the microenvironment cells perceive within living tissues, and how these change in space and time, is essential to understanding cellular mechanosensation in vivo, during normal development, tissue homoeostasis and disease. Moreover, this knowledge can help in the design of scaffolds for tissue engineering applications that better mimic not only the mechanical parameters that cells perceive in vivo, but also the characteristics of the structures responsible for that mechanical response.

## Methods

### Zebrafish husbandry, fish lines and labelling

Zebrafish (*Danio rerio*) were maintained as previously described^51^. Experiments were performed following all ethical regulations and according to protocols approved by the Institutional Animal Care and Use Committee (IACUC) at the University of California, Santa Barbara. For ubiquitous labelling of cell membranes we used *Tg(actb2:MA-Citrine)* embryos^52^ or embryos injected at the one-cell stage with membrane-GFP messenger RNA (mRNA; GFP, green fluorescent protein). For ubiquitous nuclear labelling and mosaic membrane labelling, wild-type (AB) embryos were injected with 40–50 pg H2B-RFP (histone H2B red fluorescent protein) mRNA at the one- to two-cell stage, and then injected with 8–10 pg membrane-GFP mRNA at the 16- to 32-cell stage. For experiments with the mosaic membrane and nuclear labelling, wild-type (AB) embryos were co-injected with 15–25 pg each of membrane-GFP and H2B-RFP mRNAs into the cells at the 16- to 32-cell stage. For N-cadherin quantification, we used *TgBAC(cdh2:cdh2-tFT)* embryos^53^. For actin dynamics quantification at cell junctions, we used *Tg(actb1:GFP-Has UTRN)e116)* (ref. ^54^) × *Tg(actb2:MA-mCherry2)hm29* (ref. ^55^) double transgenic embryos. For myosin dynamics quantification at cell junctions, we used *Tg(actb2:myl12.1-EGFP)* (ref. ^56^) × *Tg(actb2:MA-mCherry2)hm29* double transgenic embryos.

### Generation and injection of ferrofluid droplets

Ferrofluid droplets were prepared as previously described^28^. Briefly, DFF1 ferrofluid (Ferrotec) was diluted in filtered 3M Novec 7300 fluorocarbon oil (Ionic Liquid Technologies) at varying concentrations to tune the saturation magnetization of the ferrofluid, thereby allowing variations in droplet deformations (applied strains) for the same applied value of the magnetic field. To prevent non-specific adhesion between cells and droplets, a fluorinated Krytox-PEG(600) surfactant (008-FluoroSurfactant, RAN Biotechnologies^57^; PEG(600), polyethylene glycol with average molecular weight of 600) was diluted in the ferrofluid at a 2.5% (w/w) concentration. The ferrofluid was calibrated before each experiment as previously described^28^, so that the applied magnetic stresses are known, enabling quantitative experiments. The droplets were directly generated inside the embryos, by micro-injection of the ferrofluid oil in the tissue of interest, as previously described^28^. The required droplet size (of average radius ~20 μm) was achieved by modulating the injection pressure and the injection pulse interval. Droplets were injected in the MPZ at the four-somite and six-somite stages for measurements in the PSM and MPZ, respectively. Experiments were performed at least 2 hours after injection to allow tissue recovery.

### Imaging

Embryos at the ten-somite stage were mounted in 0.8% low-melting agarose and imaged at 25 °C using a laser scanning confocal microscope (LSM 710, Carl Zeiss) running the software Zen 2012 sp5 (Carl Zeiss). Confocal images of the region of interest in ubiquitous or mosaic membrane-labelled embryos were taken either at 2.5 s intervals using a ×40 water immersion objective (LD C-Apochromat 1.1 W, Carl Zeiss) or through a 3D timelapse with 1.0 µm optical sections every 16 s using a ×25 water immersion objective (LD C-Apochromat 1.1 W, Carl Zeiss). Imaging of ferrofluid droplets in the embryo was done as previously described^28^. To visualize myosin and actin dynamics at the cell–cell contact over time, confocal images of *Tg(actb2:MA-mCherry2)hm29* × *Tg(actb2:myl12.1-EGFP)* and *Tg(actb2:MA-mCherry2)hm29* × *Tg(actb1:GFP-Has UTRN)e116)* double transgenic embryos, respectively, were taken in the region of interest at 2 s intervals using a ×40 water immersion objective. Ferrofluid droplets were labelled using a custom-synthesized fluorinated rhodamine dye^58^, which was diluted in the ferrofluid oil at a final concentration of 37 μM.

### Magnetic actuation of ferrofluid microdroplets

Actuation of ferrofluid droplets was performed following the previously described protocol^28^. Briefly, a ferrofluid droplet embedded in the tissue was deformed by applying a uniform and constant magnetic field^28^. The magnetic field was applied for 15 minutes, as this timescale is longer than typical cellular processes but minimal tissue rearrangements due to tissue morphogenesis occur within this time period. After 15 minutes the magnetic field was removed and the droplet was monitored during relaxation for an additional 15 minutes, as droplet relaxation occurs over much shorter timescales (Fig. 1d). Upon application of a uniform, constant magnetic field, ferrofluid droplets acquire an ellipsoidal shape, elongated along the direction of the applied magnetic field^28^. In our experiment we monitored the time evolution of the droplet deformation (by quantifying the ellipsoidal shape) and measured the initial (*e*_0_), maximal (*e*_M_) and residual (*e*_R_) droplet elongation in the direction of the applied magnetic field.

### Determination of junctional lengths and their dynamics

To monitor cellular junctions over time, we acquired confocal sections of embryos injected with membrane-GFP mRNA every 5 s for a total period of 30 minutes. The location of cells’ vertices and junctional lengths in the images were detected using Tissue Analyzer^59^. For each embryo, we segmented a region of interest (ROI) along the AP axis for which cell–cell contacts were trackable over a period of at least 400 s. We then used the Tissue Analyzer package to obtain the time evolution of contour lengths of cell–cell contacts and the (*x*, *y*) positions of the vertices. The average junction length $\bar \ell$ is an ensemble average over different junctions in the tissue.

### Normalized frequency (distribution) of junctional length variations (endogenous strains)

For each one of the time series of junctional length (obtained as described in the section ‘Determination of junctional lengths and their dynamics’), we obtained the time average of the junctional length over a period of 400 s, namely $\bar \ell _t$, and calculated the relative deviations of the junctional length from its average, namely $\left( {\ell - \bar \ell _t} \right)/\bar \ell _t$. Since actomyosin activity at the cell junctions in known to drive the dynamics of cell junctions, these actively generated relative changes in junctional length are a measure of the local, endogenous applied strains. We analysed four ROIs in both A-PSM and P-PSM, and three ROIs in the MPZ, all corresponding to different wild-type embryos. We obtained the normalized lengths $\left( {\ell - \bar \ell _t} \right)/\bar \ell _t$ over the time window of a single experiment and combined, for each region, all the values of the strain magnitude (absolute value of $\left( {\ell - \bar \ell _t} \right)/\bar \ell _t$ or $\left| {\left( {\ell - \bar \ell _t} \right)/\bar \ell _t} \right|$), for all times and cell–cell contacts into a single, normalized frequency distribution $p\left( {\left| {\left( {\ell - \bar \ell _t} \right)/\bar \ell _t} \right|} \right)$ of the endogenous strain magnitude $\left| {\left( {\ell - \bar \ell _t} \right)/\bar \ell _t} \right|$.

### Determination of protrusion lengths and their dynamics

To monitor cell protrusions over time, we acquired time lapses (for 12 min or 18 min at one frame every 2.5 s or 16 s, respectively) of confocal sections of each region of the tissue (MPZ, P-PSM and A-PSM) in wild-type embryos with mosaic membrane labelling. Since only a subset of cells were labelled, cell protrusions between cells were visible. Tracking of cell protrusion length over time, namely $\ell _{\mathrm{p}}(t)$, was done manually using ImageJ for each protrusion. The segmented line tool was used to follow each protrusion path, with a large enough width to include the protrusion. The locations of the tip and base (origin of the protrusion on the cell) of each protrusion for each time point were determined from the sharp fluorescence changes along the segmented path. The length of the protrusion was then determined for each time point as the difference in length between the tip and base of the protrusion.

### Protrusion strain

To calculate the maximal strains that protrusions can generate, we first determined the maxima in protrusion lengths, namely $\ell _{\mathrm{p}}^{\mathrm{M}}$, for each protrusion. We excluded maxima separated by less than 0.5 µm or with a protrusion length smaller than 0.5 µm because this was close to our spatial resolution. For each protrusion, we then obtained the amplitude of its maximal variations, namely $\ell _{\mathrm{p}}^{\mathrm{M}} - \bar \ell _{\mathrm{p}}$, where $\bar \ell _{\mathrm{p}}$ corresponds to the average protrusion length. Since our experimental data showed that the relevant characteristic length scale controlling the onset of plasticity is the junctional length, we obtained the maximal strain applied by protrusions by calculating the ratio of the maximal length variations of protrusions and the average junctional length, namely $\left( {\ell _{\mathrm{p}}^{\mathrm{M}} - \bar \ell _{\mathrm{p}}} \right)/\bar \ell$. The actual applied strains will, in general, be smaller than the measured values of maximal strains reported here, as the protrusions did not retract all their length instantaneously.

### Protrusion shear rate

Once the time evolution of the protrusion length $\ell _{\mathrm{p}}(t)$ was determined, we calculated the instantaneous shear rate as $\left( {1/\ell _{\mathrm{p}}} \right){\mathrm{d}}\ell _{\mathrm{p}}/{\mathrm{d}}t$. We applied a B-spline using Mathematica (Wolfram, v.13.1.0.0) to the measured protrusion length before calculating ${\mathrm{d}}\left( {\ln \ell _{\mathrm{p}}(t)} \right)/{\mathrm{d}}t$ in Matlab (MathWorks, v.2020b). This derivative provides the instantaneous shear rates.

### Measurement of relaxation timescales and (linear) mechanical properties of the microenvironment

Embryos with a previously injected ferrofluid droplet were mounted for imaging and inspected on the confocal microscope, as described above in ‘Magnetic actuation of ferrofluid microdroplets’. Briefly, after the droplet was located, we lowered the magnet array to the distance from the sample that generates the desired magnetic field (and magnetic stress) to create only small droplet deformations leading to applied strains within the 5–20% range, well below the observed yield strain (occurring at 100%). The magnet array was kept at this position for 15 minutes and then moved away from the sample, leading to droplet relaxation. The ferrofluid droplet was imaged for the entire actuation cycle, enabling segmentation of the droplet’s shape in each frame and quantification of deformation dynamics. To obtain all the mechanical parameters in the rheological model (Fig. 2a), we used a previously developed software^28^, which fits the time evolution of the droplet deformation to the mathematical solutions of the rheological model. The mechanical parameters in the rheological model are obtained from the fit parameters, as previously described^28^. Since the droplet capillary stress acts effectively as an elastic element acting in parallel on all branches in the generalized Maxwell model^28^, the elastic element associated with the supracellular tissue stiffness (branch 3 in Fig. 2a) and the droplet capillary stress cannot be obtained independently by applying strains below yield. To decouple them, we obtained the effective elastic contribution of the droplet capillary stresses from measurements above yield, as in this case the elastic component of the tissue is not present at long timescales because stresses in the tissue relax via cellular rearrangements. Once the contribution of the capillary stress was known, we removed it from the long-timescale elasticity below yield (branch 3 in Fig. 2a). Importantly, the mechanical properties measured with this technique correspond to a local average of the mechanical properties characterizing the surrounding material, along different spatial directions.

### Visualization of extracellular space

To visualize the volume fraction of the extracellular spaces, we injected Dextran, Alexa Fluor 488 (molecular weight, 10,000) in the MPZ of nine-somite stage embryos. After 30–45 minutes, embryos were mounted and imaged as described above.

### N-cadherin measurements

To visualize the levels of N-cadherin (cdh2) in the paraxial mesoderm, we used the transgenic line *TgBAC(cdh2:cdh2-tFT)* (ref. ^53^), a previously established reporter line for N-cadherin. The density of N-cadherin along the AP axis was measured from a confocal section through the paraxial mesoderm. First, a line along the AP axis through the middle of the paraxial mesoderm was defined. We the measured the average N-cadherin signal on a band around the line of thickness 2 μm (to visualize cell-to-cell variation along the AP axis) and another of 60 μm (to average out cell-to-cell variations along the AP axis).

### Persistence timescale of junctional length dynamics

Using the time series of the contour length of cell junctions (obtained as described in the section ‘Determination of junctional lengths and their dynamics’), we calculated the temporal autocorrelation function, namely

$$C_{\ell \ell }\left( \tau \right) \equiv \frac{{\left\langle {\left( {\ell \left( {t + \tau } \right) - \left\langle {\ell \left( {t + \tau } \right)} \right\rangle _t} \right) \left( {\ell \left( t \right) - \left\langle {\ell \left( t \right)} \right\rangle _t} \right)} \right\rangle _t}}{{\sqrt {\left\langle {\left( {\ell \left( {t + \tau } \right) - \left\langle {\ell \left( {t + \tau } \right)} \right\rangle _t} \right)^2} \right\rangle _t} \sqrt {\left\langle {\left( {\ell \left( t \right) - \left\langle {\ell \left( t \right)} \right\rangle _t} \right)^2} \right\rangle _t} }}$$

where $\ell$ is the junctional length of any given cell–cell junction, *τ* is the time shift and $\left\langle \ell \right\rangle _t$ is the time average of a given junctional length. Time averages are used here because we are interested in the autocorrelation for a given junction. To reduce the numerical errors that result from using time averages, these were calculated using time-shifted intervals; that is, each average $\left\langle \cdot \right\rangle _t$ was evaluated with the data in a time interval (0, *T* − *τ*), with *T* being the duration of the experiment. We analysed each ROI and obtained the correlation function for each embryo separately. The obtained autocorrelation functions were nearly exponential in all cases. The persistence timescale of the junctional dynamics corresponds to the autocorrelation timescale, which we obtained by fitting an exponential function to the autocorrelation decay. The reported characteristic timescale was obtained from a weighted average of the timescales measured in different embryos, with the weights being the inverse of their variances.

### Fourier transform of junctional length dynamics

We calculated the Fourier transform of the junctional length $\ell$ (obtained as described in the section ‘Determination of junctional lengths and their dynamics’) using the discrete fast Fourier transform of the measured junctional-length time series. To remove the effect of supracellular, long-timescale tissue movements on the junctional lengths, we first filtered the junctional-length time series using a high-pass filter with a cut-off frequency of approximately 1/6 min^−1^. This suppresses junctional-length fluctuations at timescales equal to or larger than 6 minutes. We checked that our results were unchanged by varying the cut-off frequency within a reasonable range. We then subtracted the time average of the filtered signal, namely $\bar \ell _t^{\mathrm{f}}$, from the time series and calculated the fast Fourier transform on the processed signal, namely $\ell ^{\mathrm{f}} - \bar \ell _t^{\mathrm{f}}$.

### Characteristic timescales of actin and non-muscle myosin II dynamics at cell–cell contacts

Tracking of the myosin–actin signal intensity along the junctions in PSM and MPZ regions (*N* = 12, two embryos) was performed using ImageJ (v.1.53i). For each junction, the average junctional intensity, *I*(*t*), was calculated at each time point and the temporal autocorrelation was computed, namely

$$C_{II}(\tau ) = \frac{{\left( {I\left( {t + \tau } \right) - < {I\left( {t + \tau } \right)} > _t} \right) \left( {I\left( t \right) - < {I\left( t \right)} > _t} \right)}}{{\sqrt {\left( {I\left( {t + \tau } \right) - < {I\left( {t + \tau } \right)} > _t} \right)^2} \sqrt {\left( {I\left( t \right) -< {I\left( t \right)} > _t} \right)^2} }}.$$

The autocorrelations were then averaged per condition (either myosin or actin) and per region (either MPZ or PSM).

### Active foam simulation of junctional fluctuations

We adapted the recently developed Active Foam theoretical framework^44^ to study the relation between actomyosin-generated tension dynamics at cell–cell contacts and the resulting cell–cell contact length fluctuations. A confluent system of 36 cells in a square periodic box was generated for an initial configuration. We scanned the parameter space of a tension persistence timescale *τ*_T_ ranging from 1 to 10 at unity increments with a fixed magnitude of the tension fluctuations. For each tension persistence timescale, ten independent simulations were executed. Simulations ran for 30*τ*_T_ to ensure that we captured the tension dynamics at timescales longer than *τ*_T_, and also that autocorrelation functions decayed to zero within the simulation run time. The tension persistence timescale and the cell–cell contact length persistence timescale, *τ*_T_ and $\tau _\ell$, respectively, were computed from an exponential fit to the tension and the cell–cell contact length autocorrelation functions, respectively.

### Statistics

In experiments involving zebrafish embryos, no statistical methods were used to predetermine sample sizes, but our sample sizes are similar to those reported in previous publications^27,28,44^. No samples were excluded from the analysis. Analysis of all the data was done by automated software to ensure blinding and avoid biases in the analysis. No randomization of the data was used. One-sample two-tailed *t*-tests were used for statistical analysis; for these, data distribution was assumed to be normal, but this was not formally tested.

### Reporting summary

Further information on research design is available in the Nature Portfolio Reporting Summary linked to this article.

## Online content

Any methods, additional references, Nature Portfolio reporting summaries, source data, extended data, supplementary information, acknowledgements, peer review information; details of author contributions and competing interests; and statements of data and code availability are available at 10.1038/s41563-022-01433-9.

## Supplementary information

Reporting Summary

## Data Availability

Source data are provided with this paper.

## Source data

Source Data Fig. 1 Datasets associated with each panel in the figure.
Source Data Fig. 2 Datasets associated with each panel in the figure.
Source Data Fig. 3 Datasets associated with each panel in the figure.
Source Data Fig. 4 Datasets associated with each panel in the figure.

## Code availability

The custom-made simulation code used in this article is publicly available on GitHub at https://github.com/campaslab/active_foam_junctional_fluctuation.
