# Supplementary material for: Mechanics of the cellular microenvironment as probed by cells in vivo during zebrafish presomitic mesoderm differentiation
Source: Nat Mater. 2022 Dec 28;22(1):135–43. doi: 10.1038/s41563-022-01433-9 (PMC9812792; doi:10.1038/s41563-022-01433-9)
Supplement: Supplementary file 1 — Reporting Summary [file 41563_2022_1433_MOESM1_ESM.pdf]

nature research

Corresponding author(s): Otger Campas

Reporting Summary

Nature Research wishes to improve the reproducibility of the work that we publish. This form provides structure for consistency and transparency in reporting. For further information on Nature Research policies, see Authors & Referees and the Editorial Policy Checklist.

Statistical parameters

When statistical analyses are reported, confirm that the following items are present in the relevant location (e.g. figure legend, table legend, main text, or Methods section).

n/a	Confirmed	
☐	☒	The exact sample size (n) for each experimental group/condition, given as a discrete number and unit of measurement
☐	☒	An indication of whether measurements were taken from distinct samples or whether the same sample was measured repeatedly
☐	☒	The statistical test(s) used AND whether they are one- or two-sided<br>*Only common tests should be described solely by name; describe more complex techniques in the Methods section.*
☒	☐	A description of all covariates tested
☐	☒	A description of any assumptions or corrections, such as tests of normality and adjustment for multiple comparisons
☐	☒	A full description of the statistics including central tendency (e.g. means) or other basic estimates (e.g. regression coefficient) AND variation (e.g. standard deviation) or associated estimates of uncertainty (e.g. confidence intervals)
☐	☒	For null hypothesis testing, the test statistic (e.g. F, t, r) with confidence intervals, effect sizes, degrees of freedom and P value noted<br>*Give P values as exact values whenever suitable.*
☒	☐	For Bayesian analysis, information on the choice of priors and Markov chain Monte Carlo settings
☒	☐	For hierarchical and complex designs, identification of the appropriate level for tests and full reporting of outcomes
☒	☐	Estimates of effect sizes (e.g. Cohen's d, Pearson's r), indicating how they were calculated
☐	☒	Clearly defined error bars<br>*State explicitly what error bars represent (e.g. SD, SE, CI)*

Our web collection on statistics for biologists may be useful.

Software and code

Policy information about availability of computer code

Data collection	Commercial Zeiss Zen software (Zeiss Zen 2012 sp5) was used to perform confocal imaging. Software to perform magnetic actuation of droplets was previously published in Ref. 28.
Data analysis	Different analysis software was used to analyze the data: Tissue Analyzer (previously published; ref. 59) was used to measure cell-cell contact lengths. Custom software to analyze the mechanical properties of the tissue from droplet deformations was previously published in Ref. 28. Mathematica (Wolfram, version 13.1.0.0), Matlab (MathWorks, version 2020b) and ImageJ (v1.53i) were also used to analyze the data.

For manuscripts utilizing custom algorithms or software that are central to the research but not yet described in published literature, software must be made available to editors/reviewers upon request. We strongly encourage code deposition in a community repository (e.g. GitHub). See the Nature Research guidelines for submitting code & software for further information.

Data

Policy information about availability of data

All manuscripts must include a data availability statement. This statement should provide the following information, where applicable:

- Accession codes, unique identifiers, or web links for publicly available datasets
- A list of figures that have associated raw data
- A description of any restrictions on data availability

All data supporting our findings are provided with this manuscript as source data files.

Field-specific reporting

Please select the best fit for your research. If you are not sure, read the appropriate sections before making your selection.

☒ Life sciences ☐ Behavioural & social sciences ☐ Ecological, evolutionary & environmental sciences

For a reference copy of the document with all sections, see nature.com/authors/policies/ReportingSummary-flat.pdf

Life sciences study design

All studies must disclose on these points even when the disclosure is negative.

Sample size	In experiments involving zebrafish embryos, no statistical methods were used to pre-determine sample sizes but our sample sizes are similar to those reported in previous publications (Refs. 27, 28, 44).
Data exclusions	No data was excluded.
Replication	We have indicated in the manuscript when specific experiments were independently replicated.
Randomization	No randomization of the data was used.
Blinding	Analysis was done by automated software which was blind to data collection. Team members doing the analysis were different from those who collected the data.

Reporting for specific materials, systems and methods

Materials & experimental systems

n/a	Involved in the study
☒	☐ Unique biological materials
☒	☐ Antibodies
☒	☐ Eukaryotic cell lines
☒	☐ Palaeontology
☐	☒ Animals and other organisms
☒	☐ Human research participants

Methods

n/a	Involved in the study
☒	☐ ChIP-seq
☒	☐ Flow cytometry
☒	☐ MRI-based neuroimaging

Animals and other organisms

Policy information about studies involving animals; ARRIVE guidelines recommended for reporting animal research

Laboratory animals	Zebrafish (Danio rerio) embryos were used in this study. Sex-specific experiments were not necessary, as zebrafish embryos at the studies stage have not yet undergone sex determination.
Wild animals	None.
Field-collected samples	None.
